# Does tumorigenesis select for or against mutations of the DNA repair-associated genes BRCA2 and MRE11?: Considerations from somatic mutations in microsatellite unstable (MSI) gastrointestinal cancers

**DOI:** 10.1186/1471-2156-7-3

**Published:** 2006-01-17

**Authors:** Michiel S van der Heijden, Jonathan R Brody, Elhaam Elghalbzouri-Maghrani, Malgorzata Z Zdzienicka, Scott E Kern

**Affiliations:** 1 From the department of Oncology, The Johns Hopkins University School of Medicine, Baltimore, Maryland; 2 From the department of Pathology, The Johns Hopkins University School of Medicine, Baltimore, Maryland; 3 Department of Internal Medicine, Academic Medical Center, Amsterdam, The Netherlands; 4 Department of Toxicology, Leiden University Medical Center, Leiden, The Netherlands; 5 Department of Molecular Cell Genetics, Collegium Medicum, N. Copernicus University, Bydgoszcz, Poland

## Abstract

**Background:**

The BRCA2 and MRE11 proteins participate in the repair of double-strand DNA breaks by homologous recombination. Germline *BRCA2 *mutations predispose to ovarian, breast and pancreatic cancer, while a germline *MRE11 *mutation is associated with an ataxia telangiectasia-like disorder. Somatic mutations of *BRCA2 *are rare in typical sporadic cancers. In tumors having microsatellite instability (MSI), somatic truncating mutations in a poly [A] tract of *BRCA2 *are reported on occasion.

**Results:**

We analyzed gastrointestinal MSI cancers by whole gene *BRCA2 *sequencing, finding heterozygous truncating mutations in seven (47%) of 15 patients. There was no cellular functional defect in RAD51 focus-formation in three heterozygously mutated lines studied, although other potential functions of the BRCA2 protein could still be affected. A prior report of mutations in primary MSI tumors affecting the IVS5-(5–15) poly [T] tract of the *MRE11 *gene was confirmed and extended by analysis of the genomic sequence and protein expression in MSI cancer cell lines. Statistical analysis of the published *MRE11 *mutation rate in MSI tumors did not provide evidence for a selective pressure favoring biallelic mutations at this repeat.

**Conclusion:**

Perhaps conflicting with common suspicions, the data are not compatible with selective pressures during tumorigenesis promoting the functional loss of *BRCA2 *and *MRE11 *in MSI tumors. Instead, these data fit closely with an absence of selective pressures acting on *BRCA2 *and *MRE11 *gene status during tumorigenesis.

## Background

The mismatch repair genes are genomic caretakers that function most visibly in the repair of simple nucleotide repeats. Neoplastic cells with defects in this pathway have a particular type of genetic instability resulting in elevated rates of sequence mutations and very high rates of mutations in microsatellites (simple repetitive sequences); hence the term microsatellite instability (MSI). Cancers displaying MSI usually harbor mutations in at least some of the specific genes that contain microsatellites in their coding sequence, such as *TGFBR2*, *BAX*, *ACVR2*, *IGF2R, MSH3 *and *MSH6 *[1,2]. Because the MSI phenotype is fully adequate to explain the accumulation of new mutations in these genes, the mere presence of the mutations does not imply that the genes have any special role in tumorigenesis. High mutation rates of a repetitive sequence in a particular gene could be caused by the length of the repeat as well as the adjacent sequence (which may allow slippage during DNA replication) and are not necessarily the result of selective pressure acting on these genes during tumorigenesis [3,4]. An exception exists for a few genes, in particular the *TGFBR2*, *ACVR2*, and *BAX *genes, where the prevalence rate (as contrasted with the mere presence) of homozygous mutations and biallelic compound mutations has been statistically demonstrated to exceed the rate expected by the chance acquisition of the two individual mutant alleles [5]. For these latter genes, one can infer that during tumorigenesis, selective pressures favored the emergence of subclones having gained a biallelic mutation. Specifically, we can justifiably refer to *TGFBR2*, *ACVR2*, and *BAX *as tumor-suppressor genes. In the cases of *TGFBR2 *and *ACVR2*, instances of somatic homozygous mutation affecting non-repetitive coding sequences are reported also in non-MSI tumors, those exhibiting chromosomal instability (CIN), strengthening the argument for a tumor-suppressive role for these genes [5].

The roles of the *BRCA2 *and *MRE11 *(*MRE11A*) genes in tumorigenesis are not so clear-cut. Many persons with a germline inactivating mutation of *BRCA2 *do not get cancer. There is little evidence that *BRCA2 *plays a gatekeeper role for neoplasia. We know, for instance, that unlike with germline mutations of the gatekeeper genes *APC*, *MEN1 *and *RB1*, the germline *BRCA2 *mutations do not give rise to a recognizable syndrome of multiple incipient neoplasms in any particular organ. Instead, it is possible that *BRCA2 *plays a tumorigenic role primarily late in the development of a neoplasm. In the case of one patient that had a germline *BRCA2 *mutation and a pancreatic cancer, the loss of the wildtype allele was observed only in the cancer and in the most histologically advanced of a number of neoplastic lesions studied; the histologically earlier stages of neoplasia still retained the wild-type allele [6]. This case, along with the finding of the wild-type allele among early pancreatic neoplasia in other patients having a germline *BRCA2 *mutation, supported a "late" role for *BRCA2 *mutation in tumorigenesis.

This "late" role is rationalized by the known impairment of the pace of the cell division cycle when the *BRCA2 *gene is knocked out genetically in nonneoplastic cells [7]. As one mechanistic explanation for this empirical observation, the successful transit from S phase to M phase requires the repair of a number of double-strand DNA breaks that arise normally in each cell cycle. The homologous recombination DNA repair system, which utilizes the endonuclease MRE11 and the RAD51-assembly template BRCA2, is an important means to repair such breaks after DNA replication [8,9]. In the absence of this system, normal cellular checkpoints may restrict the progress of the cell cycle. Thus the role of *BRCA2 *defects during breast, pancreatic, and ovarian tumorigenesis may be primarily to increase the genetic instability, but to do so only once the neoplastic clone has accumulated a number of defects in checkpoint functions, a situation that would exist in late, rather than early, stages of tumorigenesis.

Although mutations in *BRCA2 *do not provide a growth advantage in nonneoplastic cells, the *BRCA2 *gene is often discussed as a "tumor-suppressor" gene. If it were so, in MSI tumors one would expect to find biallelic mutations at a rate higher than expected from the individual rates of mutant alleles [5,10]. Alternatively, if *BRCA2 *were primarily serving a role in genome maintenance (rather than in growth regulation or some other proper and expected "tumor-suppressor" function), its functional loss might often be selected against. In this latter scenario, the prevalence of biallelic mutations would be found to be unexpectedly low even in the face of a high prevalence of mutant single alleles.

It would thus be especially instructive to interrogate the numerical data available from the MSI tumors; these human tumors are a form of natural long-term experiment. A heterozygous somatic mutation in an [A]_8 _tract at codon 602–605 of the *BRCA2 *gene was discovered in an ovarian MSI tumor by Takahashi et al [11]. This short repeat was subsequently examined by several groups: a somatic mutation prevalence rate of 0–5% in MSI cases was reported [12-15]. In a study of 12 MSI endometrial tumors by Koul et al, six somatic *BRCA2*-mutations were found in five tumors [16]; one intronic mutation identified four times, however, was later criticized as being unrelated to gene function [17]. Owing to the incomplete examination of the large *BRCA2 *gene sequence in the prior studies, the findings were inadequate to infer any tendencies towards or against the functional inactivation of *BRCA2 *that might act during tumorigenesis. Here, we study *BRCA2 *frameshift mutations in cell lines of gastrointestinal MSI tumors and provide functional testing of *BRCA2*-related cellular responses.

Briefly, we also revisited the recently reported genetic alterations affecting a mononucleotide repeat tract of the *MRE11 *gene. These mutations, like the heterozygous mutations of *BRCA2 *in MSI tumors, raise somewhat similar questions regarding the role of a mutated gene in tumorigenesis. The *MRE11 *gene encodes a protein involved in the repair of double-stranded breaks and has an intronic [T]_11 _tract four basepairs from the 5' end of exon 5, harboring nucleotide deletions in colorectal MSI cases [18]. These observed deletions were associated with expression of an altered transcript of the *MRE11 *gene, splicing out exon 5 and leading to decreased or absent MRE11 protein expression [18].

## Results and discussion

### Heterozygous BRCA2 mutations in gastrointestinal MSI cancer cells

MSI tumors have a dramatically increased rate of mutations, especially in genes containing microsatellites. Inconveniently for researchers, mutations in such tracts do not necessarily imply a causal role for the affected genes, as noted in the first study describing such alterations in human tumors [19]. Yet, if mutations in a given gene prove beneficial to the developing neoplasm, newly mutated subclones of cancer cells having acquired this mutation can outcompete other clones that do not possess the mutation. This selective pressure can be mathematically proven from the mutational prevalence rates found in human tumors, as has been done for the *TGFβRII *and *ACVRII *genes [5]. If mutations in *BRCA2 *would be positively selected (i.e., selected for) in MSI tumors, one would expect biallelic mutations in *BRCA2 *to frequently be found. Alternatively, heterozygous mutations could have a dominant-negative effect or could lead to haplo-insufficiency with some loss of function, while perhaps still providing a growth advantage to the developing cancer cell.

Upon whole gene sequencing of *BRCA2 *in 14 gastrointestinal MSI cancer cell lines and one pancreatic adenocarcinoma xenograft, a single heterozygous frameshift mutation was found in the xenograft and in each of five cell lines. Two heterozygous mutations were encountered in one additional cell line (Table 1). Four of these mutations occurred in nucleotide repeats different from those previously reported; mutations at nucleotides 1807–1813 (codons 602–605) [11] and 5355–5361 (codons1785–1787) [16] had been reported previously [9-14]. The novel MSI mutations included deletions within an [A]_7 _tract at nucleotides 5067–5073 (codons 1689–1691), an [A]_7 _tract at nucleotides 9103–9110 (codons 3035–3037), and a non-repetitive site at nucleotides 3599–3600 (codon 1200). All of these frameshift mutations are expected to result in truncation of the BRCA2 protein and abrogation of its function. The mRNA expression of four different *BRCA2*-mutations was assayed by reverse transcriptase-PCR in four cell lines: LS174T (9110delA), RKO (5361delA), MIP101 (5361delA, 3599delGT) and PL3 (5073delA); all mutant alleles were found to be expressed. In addition to the deleterious frameshift mutations, several missense variants were found (Table 1). Most of these sequence changes are known variants of the *BRCA2 *gene that do not segregate with cancer in affected families and were judged as unlikely to have an effect on BRCA2 protein function.

**Table 1:** *BRCA2 *and *MRE11 *mutations in MIN cell lines

Tumor Cell Line/Xenograft*	***Truncating BRCA2 Mutations***	***Other BRCA2 Variants***	MRE11A Mutations**
MIP101 (colorectal)	5355delA, 3599delGT	R2784G	T9/T10/T11
LS174T (colorectal)	9110delA		T9/T10
RKO (colorectal)	5355delA		T8/T9/T10
HCT116 (colorectal)	9110delA		T9/T10
KM12 (colorectal)	5355delA	K1565N	
PL3 (pancreatic)	5073delA		T9/T10
PL5 (pancreatic)	None detected		T9/T10
PX196 (pancreatic)	1813delA	S326R	
Vaco481 (colorectal)	None detected	I2944F, K1777del3	

*Six additional MSI cell lines had no truncating mutation or other variant of the BRCA2 sequence.

**Nucleotide deletions of the [T]_11 _tract. The mutations in LS174T, MIP101, and HCT116 were reported previously [18, 22, 23]

### Retention of BRCA2 function as assayed by RAD51 focus formation

**Figure 1:**
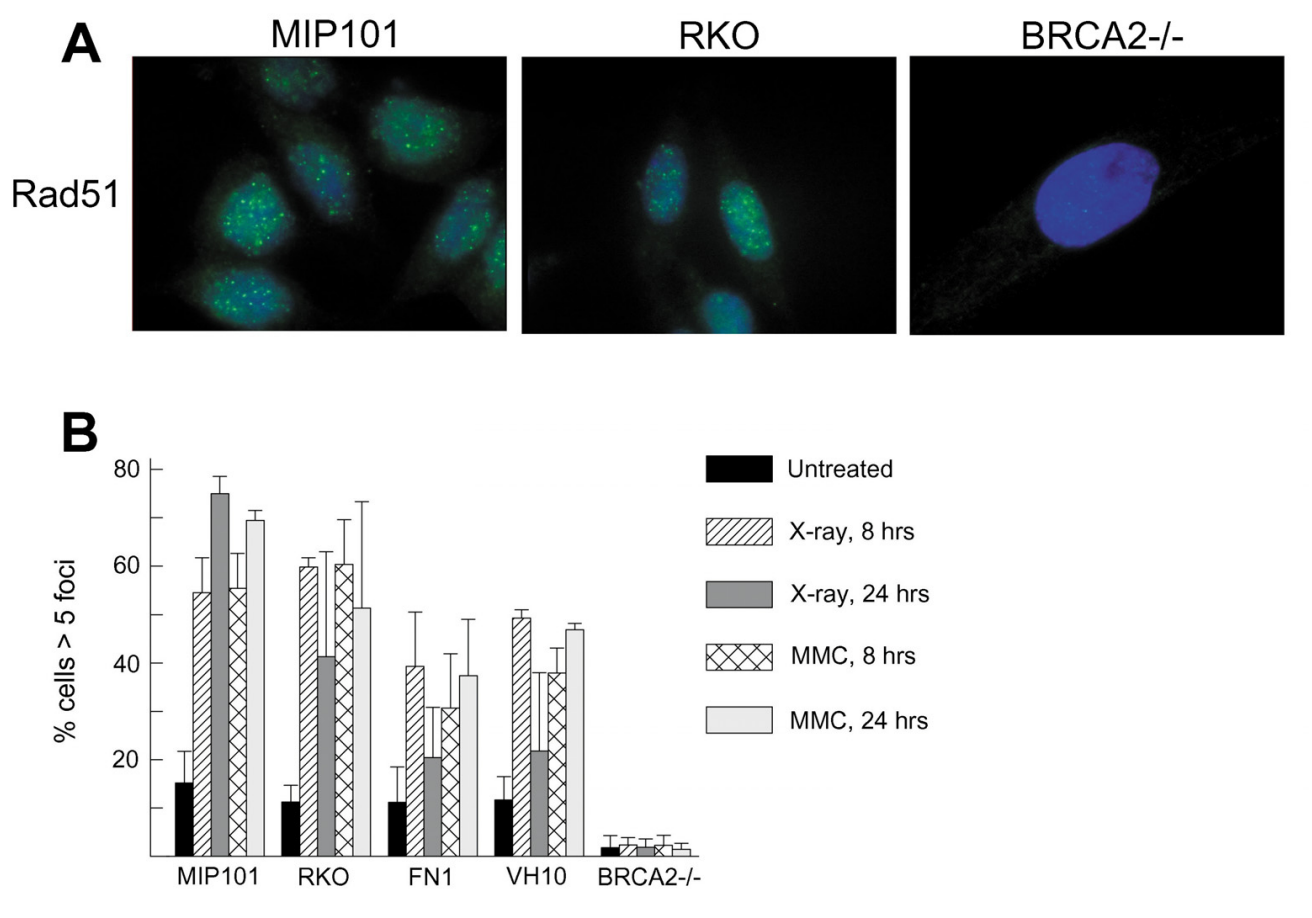
**Rad51 focus formation in *BRCA2*-mutated cell lines**. ***a. ***Cells were treated for 8 hours with MMC (2.4 μg/mL) and immunolabeled for RAD51 (green) and DAPI (blue). MIP101 and RKO are colorectal MSI cancer cells harboring *BRCA2 *frameshift mutations (see text). *BRCA2*^-/- ^cells (EUFA423/FA-D1) are derived from a Fanconi anemia patient with complementation group D1/BRCA2 ***b. ***Cells were treated with MMC (2.4 μg/mL) or ionizing radiation (12 Gy); after recovery for 8 or 24 hours, cells were stained for RAD51. RAD51 foci were counted and the percentage of cells with > 5 foci was determined. FN1 and VH10 are nonneoplastic *BRCA2-*wildtype cells.

RAD51 is a protein involved in homologous recombination that forms nuclear foci in response to DNA damage; BRCA2 function is needed for the formation of these foci [20]. To evaluate the possibility of a loss of *BRCA2*-gene function, we determined RAD51 focus-formation in response to treatment with mitomycin C and ionizing radiation in three mutated MSI cancer cell lines (PL3, MIP101 and RKO), in one MSI cancer cell line without *BRCA2 *mutations (PL5) and in two nonneoplastic *BRCA2 *wildtype cells (FN1 and VH10). None of these cell lines had a defect in RAD51 focus formation (Figure 1 and data not shown), evidence of intact BRCA2 function in all of these cell lines. The *BRCA2*-deficient cell lines CAPAN1 [21] and FA-D1 (derived from a Fanconi anemia patient with BRCA2 mutations) lacked RAD51 focus-formation and served as positive controls having functional pathway abnormalities [20]. Additionally, we determined the sensitivity of PL3, PL5, MIP101 and RKO to mitomycin C and irradiation; heterozygous mutations in the *BRCA2 *gene did not result in an increase in sensitivity to these genotoxic agents (data not shown).

### MRE11 intronic nucleotide deletions and expression in MSI cancer cells

Another DNA repair gene, *MRE11*, contains an intronic poly [T]-tract near the 5' boundary of exon 5; shortening of this repeat occurs frequently in MSI tumors. To confirm the alterations of this repeat, we sequenced the IVS5-(5–15) poly [T] tract (normally a T11) of the *MRE11 *gene in six MSI cell lines. We confirmed the reported nucleotide deletions in LS174T (T9/T10), MIP101 (T9/T10/T11), and HCT116 (T9/T10) [18,22], and uncovered additional biallelic variants in RKO (T8/T9/T10), PL3 (T9/T10), and PL5 (T9/T10) cell lines (Table 1). The finding of more than two alleles in both MIP101 and RKO could be due either to trisomy or heterogeneous cell populations. The presence of three alleles in MIP101 is a confirmation of the study by Giannini et al. [18] and is therefore likely to be caused by trisomy rather than the presence of different cell populations. By MRE11 immunoblot, we confirmed full-size MRE11 protein as being expressed in all cell lines (data not shown). MIP101 had heterozygous mutations affecting two of the three alleles and thus retained a wildtype allele of the *MRE11 *gene; MRE11 protein was expressed at a higher level in MIP101 than in cell lines having all alleles mutated (data not shown). The MRE11 protein is still expressed in these cell lines, however, and although possible disturbances in the MRE11/NBS1/RAD50 complex have been shown by Giannini et al. [23], an unequivocal defect in MRE11 function has not yet been shown. An indirect argument in favor of some retained function was provided by a demonstration that MRE11 was essential for maintenance of chromosomal DNA in vertebrate cells [9]. There is also a report of MRE11 binding to the MLH1 protein (a member of the mismatch repair system), which if significant might complicate the MRE11 functional testing in some MSI tumors [24]. Additional functional assessment of the MRE11 protein in MSI tumors was beyond the scope of this study.

### Statistical analysis of mutational prevalence rates

We observed the following prevalence rates of *BRCA2 *sequences: biallelic wildtype, 53.3%; heterozygous, 46.7%; biallelic mutant, 0%. Given the assumptions under the theory of quantitative selection constants (QSC) [5,10] and deriving the expectations solely from this rate of biallelic wildtype sequences, the expected heterozygous mutation prevalence would be 39.4% and the biallelic mutant prevalence due to a chance distribution of mutations among the two alleles (i.e., under the null hypothesis) would be 7.3%. According to the binomial test for QSC data and N = 15 cases, the likelihood (binomial sum) for the absence of biallelic mutations in this panel was 0.331. An examination of the statistical power, given a 53.3% prevalence of homozygous wildtype alleles and an observed 0% rate of homozygous mutant tumors, showed that 40 MSI tumors would be the minimal number required to produce a binomial sum of less than 0.05. Thus, the number of cases needed to evaluate the *BRCA2*-related selective pressures (one would be advised to use a multiple of the minimal necessary number) vastly exceeded the number of MSI cell lines available (15 MSI cell lines were available). Numerical arguments such as those considered here would benefit from higher numbers of analyzed tumors. Attaining such numbers in an independent larger tumor panel would be difficult: to be able to functionally test tumors with *BRCA2 *mutations, cell lines would be needed. However, the number of known MSI cancer cell lines is currently limited.

Another argument for gastrointestinal selection against deficiency of the *BRCA2 *gene comes from a recent report by Hay et al., studying mice in which *BRCA2 *was biallelically mutated in the small intestine [25]. A p53-dependent increase in apoptosis was noted, ultimately resulting in the removal of BRCA2-deficient cells, perhaps protecting against tumorigenesis. Although this study was not done in the setting of an MSI background, it does support the hypothesis that the loss of BRCA2 function is often selected against.

A prior report provided the prevalence rates of *MRE11 *mutations in colorectal cancer: biallelic wildtype, 16.3%; heterozygous mutant, 44.9%; biallelic mutant, 38.8% [23]. Given this rate of biallelic wildtype sequences, the expected heterozygous mutation prevalence would be 48.1% and the expected biallelic mutant rate due to a chance distribution of mutations among the two alleles would be 35.6%. The observed and expected rates are highly concordant. The null hypothesis, that selection pressures during tumorigenesis had not acted to influence the ratio between heterozygous (i.e., non-inactivating) and homozygous (inactivating) mutations, was not rejected and indeed appeared to explain the observed data rather well. Prior studies of human MSI cancer cell lines demonstrated that the prevalence rate of mutations was positively correlated with the length of the repeat [26]. The most complete of these studies did not examine repeats longer than an eight mono-nucleotide run, but it could reasonably be extrapolated that an 11-mononucleotide run, such as that present in the *MRE11 *gene, would have a mutational prevalence rate much higher than 50% of MSI tumors even in the absence of selective pressures favoring mutations of a given gene. These prior authors noted, however, that there was a strong and unexplained contextual variation in mutational prevalence rates, and thus the mere prevalence rate of the mutations cannot directly offer evidence for or against selective pressures having acted during tumorigenesis upon the functional status of a given gene.

In 2001, Fukuda et al. discovered three missense mutations in the *MRE11 *gene in two breast carcinomas and one lymphoma [27]. At least one of these three mutations was somatic; it is unclear whether these mutations occurred in MSI defective tumors. Wang et al. recently discovered eight somatic mutations in the *MRE11 *gene to be present in seven colorectal CIN cancers (168 tumors were studied), a rate not easily explained by chance alone, as random somatic mutations in non-MSI cancers are very rare [28]. Further studies are warranted to investigate the role of functional MRE11 defects in the development of CIN and MSI tumors, but the mutational data do not yet support a role in MSI cancer cells.

## Conclusion

We found heterozygous frameshift mutations in the *BRCA2 *gene to be present in 47% of gastrointestinal MSI cancer cell lines, a much higher percentage than previously reported. During tumorigenesis, mismatch repair deficient cancer cells evidently experiment extensively with *BRCA2 *mutations, perhaps as a consequence of the high number of repetitive sequences in the *BRCA2 *gene in combination with locus susceptibility to mutation. No impairment in BRCA2 function as assayed by RAD51 focus formation was detected, however. One cell line, MIP101, had two mutations in the *BRCA2 *gene, but no defect in RAD51 focus formation. We thus surmise that these two mutations exist in cis on one allele, with the cells retaining the other *BRCA2 *allele in wildtype form.

Although the trend is not yet statistically significant, our data raise the possibility that retention of *BRCA2 *function is selected for, and that biallelic *BRCA2 *mutation is selected against, during tumorigenesis of MSI tumors. Mutations of *MRE11 *are probably selection-neutral, and thus may not be functionally important in tumorigenesis. Our findings are not compatible with a strong selective pressure favoring abrogation of BRCA2 function in MSI tumors. Loss of BRCA2 function in addition to a defect in mismatch repair is perhaps detrimental to cell maintenance: a limited level of genetic instability could be beneficial to tumorigenesis, but excessive DNA repair defects would not. While the functional inactivation of the *BRCA2 *gene inhibited the growth of normal cells [7], it might accelerate the rate of genetic changes in developing CIN tumors. Some of these genetic changes could provide a survival advantage for an emerging subclone that would acquire a biallelic *BRCA2 *mutation. In MSI cells, however, a highly elevated level of experimentation with mutations is already present. Loss of BRCA2 function might not add much to the rate of genetic experimentation in MSI cells and could merely provide an inhibition of growth, making it unlikely that these cells could outcompete other subclones. Perhaps mutations in the *BRCA2 *gene are likely to occur only in a narrow window of opportunity, when an increase in genetic experimentation outweighs the growth inhibition caused by the loss of *BRCA2*.

## Methods

### Samples

Pancreatic cancer cell lines PL3 and PL5 were gifts of Dr. Elizabeth M. Jaffee (Johns Hopkins University). The pancreatic xenograft PX196 was established in our laboratory, as previously described [29]. Colorectal cancer cell lines LS174T and RKO, and genomic DNA for colorectal cell lines LoVo, Vaco5, Vaco6, HCT116, C, VACC1430, LIM1215, LIM2412, Vaco481 and KM12, were gifts of Drs. Bert Vogelstein and Christoph Lengauer (Johns Hopkins University). The colorectal cell line MIP101 was a gift of Dr. J. Milburn Jessup (Georgetown University). FN-1 cells are primary buccal mucosafibroblasts derived from a healthy 11 year old boy; VH10 cells are primary fibroblasts derived from the foreskin of a 10 year old healthy boy. EUFA423/FA-D1 cells are primary fibroblasts from a Fanconi anemia patient with complementation group D1 and are a kind gift of Dr. H. Joenje (Departement of Clinical and Human Genetics, Free University Medical center, Amsterdam, The Netherlands).

### Sequencing

*BRCA2 *sequencing was performed by Myriad Genetics Inc. (Salt Lake City, Utah). Primers for RT-PCR and confirmatory genomic sequencing were obtained from IDT-DNA (Coralville, Iowa). The poly [T] tract in the *MRE11 *gene was analyzed by automated sequencing of PCR-amplified genomic DNA. Prevalence rates of the mutations were analyzed by the techniques described previously [5,10].

### Immunofluorescence labeling and microscopy

To examine RAD51 foci formation, cells were grown on sterile glass slides for two days, giving sub-confluent cells at time of fixation. Then, cells were either mock-treated or treated with MMC (2.4 μg/ml for 1 h) or irradiated with 12 Gy of X-ray. After an 8 or 24 h recovery period, cells were fixed with 2% formaldehyde in PBS and permeabilized for antibody staining with PBS/0.1% Triton X-100. Subsequently the slides were blocked for 15 min in PBS/BSA (0.5%)/glycin (0.15%) and thereafter incubated with rabbit anti-HsRAD51 antiserum (FBE2) for 90 min at 37°C in a humidified atmosphere. The slides were washed three times in PBS/0.1% Triton X-100 and then incubated with AlexaTM 488-conjugated goat anti-rabbit IgG (Molecular Probes) for 1 h at 37°C in a humidified atmosphere. After three washes with PBS/0.1% Triton X-100 the cells were counterstained with 4',6-diamino-2-phenylindole (DAPI; 0.15 μg/ml) in Vectashield mounting medium (Vector Laboratories). RAD51 foci were examined under a Leitz Axioplan fluorescence microscope. A cell containing more than five distinct foci was considered positive.

## List of abbreviations

MSI Microsatellite Instability

CIN Chromosomal Instability

QSC Quantitative Seletion Constants

## Authors' contributions

MSH carried out the molecular genetic studies, managed the project and drafted the manuscript. JRB carried out the immunoblots and contributed in drafting the manuscript. EE-M carried out the immunfluorescence and the survival studies. MZZ participated in the molecular biological studies and contributed in drafting the manuscript. SEK carried out the statistical analysis, participated in drafting the manuscript and supervised the project. All authors read and approved the manuscript
